# Estimating the Reliability and Validity of the Quadrant Hop Test in the Assessment of the Functional Stability of the Knee

**DOI:** 10.7759/cureus.60516

**Published:** 2024-05-17

**Authors:** M Vijayakumar, Tushar J Palekar

**Affiliations:** 1 Physiotherapy, Dr. Dnyandeo Yashwantrao (DY) Patil Vidyapeeth University, Pune, IND

**Keywords:** quadrant hop test, knee instability, limb symmetry index, functional performance, hop test

## Abstract

Background

Many sporting activities demand multidirectional skills and movements, emphasizing the importance of various fitness components such as functional stability, strength, power, endurance, and range of motion. These aspects must be thoroughly assessed before athletes can return to sports safely following an injury. Although the single-leg hop test (SHT) is widely used as the gold standard for evaluating joint stability, it has limitations in assessing multidirectional movements. Therefore, further research is necessary to explore whether increasing the dynamicity of the hop test in different directions enhances its sensitivity in assessing knee joint stability across all four directions. The objective of this study was to investigate the applicability of a new functional assessment tool, the quadrant hop test (QHT), for evaluating lower limb functional stability.

Methodology

One hundred nineteen amateur sportsmen who are in the age group of 18-25 years with a limb symmetry index of the lower limb calculated through SHT of >80% were included. All the participants performed the SHT, a triple hop test (THT), a crossover hop test (CHT), and the QHT on two different days, with two investigators assessing the QHT on different days and then recording the measurements of Hop distance.

Results

The mean difference between SHT, THT, and CHT with QHT was 4.59%, with a moderate correlation between all the hop tests. The Cronbach's alpha revealed good intra-rater (0.917) and inter-rater reliability (0.912) of the QHT.

Conclusion

The QHT proves to be a reliable and valid measure for assessing the functional stability of the lower limb and is 4.59% more sensitive than SHT, THT, and CHT in assessing knee stability and in return to sports criteria.

## Introduction

Running, jumping, and direction changing at rapid speeds in a fast-paced setting are essential skills for success in sports. Sports medical professionals, such as doctors and surgeons, frequently treat athletes who have suffered injuries to their lower extremities with conservative care or surgery. Based on the amount of time that has passed since surgery and the patient's functional ability, the decision is made to permit an athlete to resume sports following an injury [1].

The final stage of the rehabilitation process concentrates on attaining a functional recovery tailored to fulfill the requirements of the athlete's sport [2]. The timetable for resumption of sporting activities following a knee injury is frequently a matter of contention and can present challenges in ascertaining. Variables such as range of motion, muscle power, discomfort levels, outcome assessments, and functional performance evaluations are typically taken into account when determining an individual's capacity to reintegrate into sports [3]. Athletes often transition back to dynamic pursuits, entailing substantial stresses on the knee joint, thereby heightening their susceptibility to potential re-injury. Consequently, it is imperative that the assessment methodologies employed to gauge a patient's preparedness for re-engagement accurately capture the requisite physical demands [4]. Any physical therapy session must document its treatment efficacy through objective, consistent, and credible measurements of functional outcomes. Reliability is essential to discerning whether variances observed during repeated assessments stem from measurement inaccuracies or signify genuine alterations in performance [4]. 

Achieving success following an injury is frequently gauged by a restoration to the same or enhanced level of functional proficiency as pre-injury. Numerous objective assessments have been outlined to determine whether someone is eligible to resume sports. These assessments typically encompass evaluations of the hamstrings-to-quadriceps (H:Q) ratio as well as tests involving single-leg hop, triple hop, crossover hop, and agility. Given that most athletic endeavors demand multidirectional movements and skills, optimizing performance often entails swift and precise execution of lateral, backward, and forward actions [4,5]. Such maneuvers are contingent upon adapting to the unpredictability of opponents or swiftly repositioning oneself to fulfill the requisite tasks. A diverse range of outcome measures is employed to evaluate these elements. Among the more frequently cited functional tests in the written works are the Star Excursion Balance Test (SEBT) and the Y-Balance test, which assess static stability, along with various hop tests and the Vail Sport Test, which gauge dynamic stability [3,6,7,8]. In SEBT, dynamic balance is assessed but not limb stability with impact loading, which was a limitation.

While the studies conducted exhibit commendable reliability and validity, they may be deficient in certain aspects. In order for a test to be considered a gold standard, it must comprehensively encapsulate the essence of the assessment of the primary aim. Consequently, the necessity to investigate whether hop tests incorporating escalating dynamism across multiple directions enhance the sensitivity in evaluating knee joint stability in numerous directions arises. Hence, the objective of the study is to establish the reliability and validity of a novel assessment tool, termed the "quadrant hop test (QHT)", designed to evaluate the overall stability of the knee joint across various directions. The test was developed by the investigator based on the gaps identified. This test focuses on establishing the reliability and validity of the new test, QHT. The new test is introduced for testing with other established hop tests to find its reliability and validity. 

## Materials and methods

The objective of the study is to establish the reliability and validity of a novel assessment tool, termed the "quadrant hop test (QHT)", designed to evaluate the overall stability of the knee joint across various directions. A total of 167 amateur sportsmen underwent initial screening, and subsequently, 119 participants were included in the study after determining their eligibility. Amateur sportsmen actively involved in outdoor sports, between the age of 18-25, of both genders, with no history of fracture, immobilization, or any medical surgical history in the past one year, BMI less than 28, were screened for inclusion in the study. After gathering demographic data and details regarding any previous injuries, among other relevant information, all the participants with LSI >/=80% through SHT were included.

Procedure

**Figure 1:**
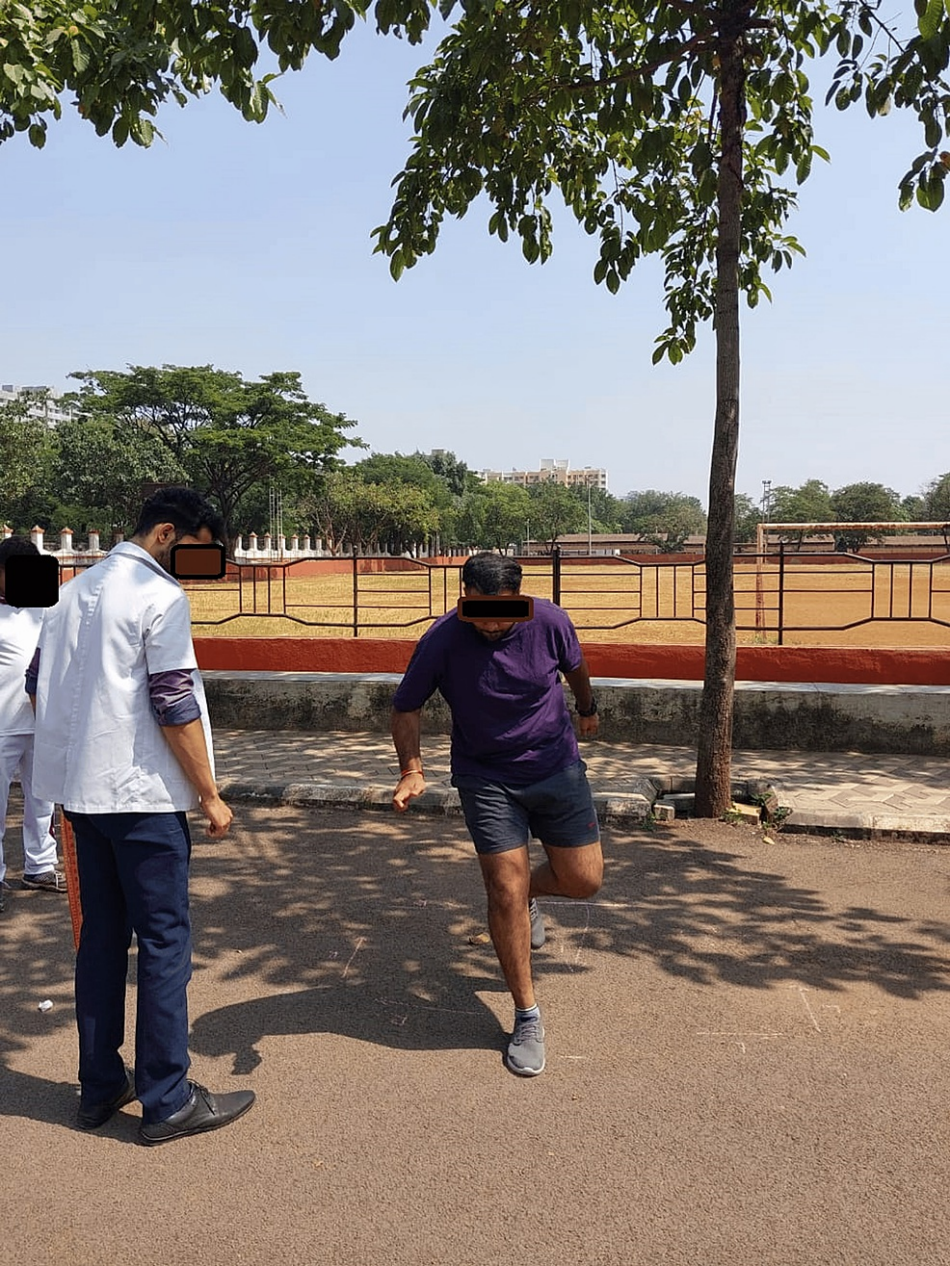
Sportsmen performing the quadrant hop test

Amateur sportsmen between the ages of 18 and 25, of both genders, with no history of fracture, immobilization, or any medical or surgical history, were screened for inclusion in the study. After gathering demographic data and details regarding any previous injuries, among other relevant information, all the participants with LSI >80% through SHT were included after obtaining their written consent. In session one, both the SHT and the new 'QHT' were explained, and demonstrated, and trials conducted. Session two involved the performance of THT, CHT, and QHT. Each test was conducted three times, and the best of the three trials of every session of every test was documented and used for analysis. In session three (i.e., 24 to 48 hours after session two), QHT was repeated for test-retest reliability. The data was recorded by investigator A for intra-rater reliability in session one and session two, and by an equivalent colleague (investigator B) for inter-rater reliability in session three. The obtained data was then analyzed for test-retest, inter-rater, and intra-rater reliability, with the analysis being conducted in a single-blinded manner. The newly designed test was compared against established procedures like the CHT, THT, and SHT to determine its validity (Figure 1).

**Figure 2:**
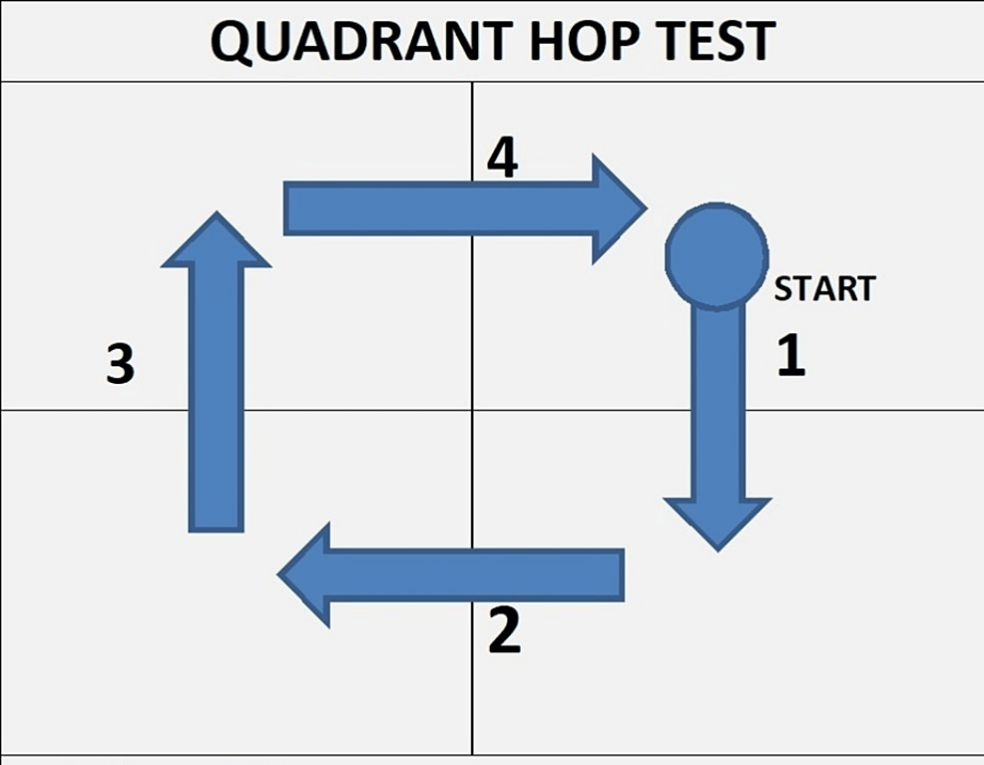
Quadrant hop test Lattice square for quadrant hop test with directions and sequence of hops 1 - forward hop; 2 - side hop (ipsilateral side / contralateral side of hop leg); 3 - backward hop; 4 - side hop (ipsilateral side / contralateral side of hop)

The proposed QHT involves the participant standing on any one leg (right or left) with their toes positioned straight forward on the beginning line. On command, a hop in the forward direction is then executed to the fullest capability, followed by hops to the right, backward, and then to the left, returning to the starting point in a quadrant direction (Figure 2). The participant must rest both legs on the ground only after completing all four hops, i.e., in all four directions. Arms are allowed to move loosely, be rested on the hips, or be tucked into the back, ensuring the same position throughout. From the beginning line to where the toe touches after landing from the hop, the total span is scaled in centimeters. Upon landing each hop, the position must be maintained for two seconds without loss of balance or additional steps. The sum of all distances in the four directions, by both legs, is calculated for Limb Symmetry Index (LSI) determination. Three trials are administered for each test, and the best distance is analyzed. Subsequently, the Limb Symmetry Index of QHT is computed and compared with the LSI obtained from other hop tests.

## Results

SPSS version 23.0 software (IBM Inc., Armonk, New York) was used to analyze the data. After the recorded data was organized, the normal distribution was examined, and the data was determined to be normally distributed. The mean age of participants was 19.910 ± 2.603 years, with a mean BMI of 27.718 ± 3.091.

The mean LSI of all the hop tests was calculated. All the existing hop tests demonstrated 94%-96% LSI, whereas QHT had 90.67% LSI with a moderate correlation coefficient. However, ANOVA between all Hop tests showed a significant difference against QHT (Table 1).

**Table 1:** Mean % of limb symmetry index of all hop tests and its correlation with QHT SHT - single hop test; THT - triple hop test; CHT - crossover hop test;  QHT( best) - best of quadrant hop; LSI - limb symmetry index

Test	N	Mean % of LSI	SD	Std. Error	ICC - r (LSI of QHT best- Pearson Correlation )	Sig. (2-tailed)	ANOVA - F value	Sig.
SHT	119	96.20	7.14	0.59	0.519	<0.0001	12.887	<0.005
THT	119	94.03	8.96	0.75	0.542	<0.0001		
CHT	119	95.54	10.87	0.90	0.513	<0.0001		
QHT (best)	119	90.67	6.92	0.57	---	---		

Correlations between the data collected by investigators A and B were used to compute the inter rater reliability. Pearson's correlation was used to determine the association between the quadrant hop test trials done on day one, day two, and day three, and the inter-rater reliability between investigators A and B for the new QHT in normal individuals by Cronbach's alpha was >0.917 (Table 2).

**Table 2:** Inter rater and intra rater reliability of QHT QHT 1 = Quadrant Hop (test by investigator A on day one); QHT 2 = quadrant hop (test by investigator A on day two); QHT 3 = quadrant hop (test by investigator B on day three)

Test	Cronbach's Alpha N of Items
QHT 1 vs. QHT 2	0.917
QHT 1 vs. QHT 3	0.957
QHT 2 vs. QHT 3	0.912

The correlation coefficient between the QHT and the SHT was found to be r=0.519 with p<0.01. The correlation between the QHT and the THT yielded a result of r=0.542 with p=<0.01. The CHT and QHT showed a r = 0.513 correlation with p = < 0.01.

The mean LSI difference between SHT and QHT is 5.53%., THT and QHT is 3.36%, and CHT and QHT is 4.87%. The LSI of QHT is less than the LSI of other hop tests by a mean of 4.59%, suggesting it is more sensitive than SHT, THT, and CHT.

The following were the findings of the inter-rater reliability: for the two items - the QHT readings obtained by investigators A and B - the Cronbach's alpha was 0.957. The Cronbach's alpha for intra-rater reliability analyzed from readings obtained by investigator A on two consecutive days, i.e., QHT 1 and QHT 2, was 0.917, thus confirming a high inter- and intra-rater reliability of the new QHT procedure.

## Discussion

The present study aimed to assess the reliability and validity of a novel multiple-directional test called the QHT, designed to evaluate the stability component of the lower extremity. This test was developed in response to the deficiency observed in previous gold standard hop tests, which omitted the inclusion of multiplanar motions [9,10]. Although hop tests are commonly regarded as objective measures that simulate the requirements of advanced sports, they might not sufficiently detect certain functional limitations due to their limited range of motion in multiple planes [11,12]. In a study conducted by Stephen Kinzey et al., all the participants balanced on one leg while reaching with the other leg in four directions. The utility of clinical diagnostic testing is dependent on the reliability and validity of the testing procedure. Because exact or near-exact repeat performances were not exhibited in this investigation, the star excursion test might not be an appropriate test of dynamic balance [9]. Recognizing these limitations, the QHT was devised to address the need for a comprehensive assessment tool capable of incorporating multiplanar movements.

The reliability of a test is a critical consideration, as it underpins the reproducibility of the test and is essential for establishing its validity. Typically, reliability is assessed using two commonly used indices: the intra-class correlation coefficient (ICC) values and the 95% confidence interval (CI) method, which have been widely recognized as appropriate measures for reliability assessment in previous literature. In this study, the results aimed at establishing reliability indicated a Cronbach's alpha of 0.957 for inter-rater reliability among normal individuals, with a significance level of p=<0.05 and a 95% confidence interval (CI). These findings demonstrate highly significant inter-rater reliability, affirming the consistency and reproducibility of the test results. The reliability of the test was established through the consistent performance of the test by two different investigators on separate days with normal individuals. This process demonstrated high reliability as both investigators adhered to the same procedure, resulting in closely aligned values for the test and yielding statistically significant outcomes.

To assess validity, the QHT was compared with the established single-leg hop for distance. While the SHT is widely considered the established standard for evaluating lower extremity stability, it lacks the crucial component of multi-directional movement, which is vital for returning to sports activities. This aspect cannot be overlooked, as it is essential for a comprehensive assessment. The choice of the tests for validation was based on the similarity between their testing components and their proven efficiency in previous studies. The correlation between the SHT and the QHT in normal individuals was 0.510 with a 95% CI, which was indicative of a positive correlation between the pre-existing SHT and the newly developed QHT in normal individuals. Similarly, the correlation between the THT and the QHT was found to be 0.542 with a 95% CI, which also indicated a positive correlation between the pre-existing triple hop test and the newly designed QHT. Also, the correlation between the CHT and the QHT was found to be 0.513 with a 95% CI. The reason for the positive correlation might be that the component of the hop tests is similar to the QHT, with additional components in the QHT. However, the correlation is found to be moderate due to the complexity of the test, which includes multi-directional hop. It was also observed that a hop in a backward direction was more complex for the participants to perform. 

A similar study was conducted by Fitzgerald et al. The results show that the described series of hop tests provide a reliable and valid performance-based outcome measure for patients undergoing rehabilitation following ACL reconstruction [7]. This assessment is notably more sensitive in gauging functional stability compared to previous tests. Its heightened sensitivity stems from its incorporation of multi-directional hopping components. Such a feature is crucial for athletes as it directly contributes to an essential aspect of athletic performance: agility. In sports, agility plays a pivotal role, demanding athletes to swiftly change direction and move effectively in various planes of motion. Thus, the inclusion of multi-directional hops in this test renders it particularly effective in evaluating an athlete's agility, a fundamental attribute for success in competitive sports [13,14].

The introduction of the QHT as a newly designed functional assessment tool represents a notable advancement in evaluating the dynamic stability of the knee. By addressing the drawbacks of standard hop tests like - a single hop test that may overestimate the performance, it is unidirectional as a forward hop only - by incorporating assessments of stability in various directions and planes, the QHT enhances the sensitivity of the evaluation process. Consequently, it can be incorporated into a comprehensive assessment battery of tests aimed at determining readiness to return to sports or meet criteria for being fit to play. This broader and more nuanced evaluation approach offered by the QHT holds promise for improving clinical decision-making and enhancing the management of athletes recovering from lower limb injuries.

There were a few limitations to the study. Firstly, it was conducted in a limited area in Pune, which meant that a broader population could not be included. Additionally, the study did not employ simple random sampling. Swift marking of Hop landing point was a difficulty. It was managed by leveling the soil for better foot prints. maintaining balance during backward hop was difficult for the players hence the trials had to repeated for perfect score.

## Conclusions

The results suggest that the QHT serves as a reliable tool for evaluating the functional stability of the knee in healthy people. Furthermore, the QHT demonstrates reliability, as evidenced by the high intra-class correlation coefficient (ICC) and the consistent reliability observed among evaluators during the test-retest procedure. Additionally, when compared to the other hop tests, the QHT offers a more comprehensive evaluation of stability in many directions, addressing earlier limitations and providing a superior measure of dynamic stability in the knee. The QHT exhibits superior construct validity, being 4.59% more sensitive than other hop tests, making it suitable for routine use by coaches and strength and conditioning specialists as part of a comprehensive assessment battery. This allows them to effectively monitor training plans for athletes, aiding in the evaluation of preparedness to return to sports or suitability for play.
